# Surgical management of gastroesophageal reflux in complex and medically fragile children: single-centre retrospective study and future perspectives

**DOI:** 10.3389/fmed.2026.1901216

**Published:** 2026-08-26

**Authors:** Anna Montemezzo, Alessandro Boscarelli, Manuela Giangreco, Elena Sofia Marcandella, Marianna Iaquinto, Edoardo Guida, Jürgen Schleef

**Affiliations:** 1 Faculty of Medicine and Surgery, University of Trieste, Trieste, Italy; 2 Department of Pediatric Surgery and Urology, Institute for Maternal and Child Health - IRCCS “Burlo Garofolo”, Trieste, Italy; 3 Clinical Epidemiology and Public Health Research Unit, Institute for Maternal and Child Health - IRCCS “Burlo Garofolo”, Trieste, Italy

**Keywords:** autologous fat grafting, children, future perspectives, gastroesophageal reflux disease, surgical treatment

## Abstract

**Introduction:**

Gastroesophageal reflux disease (GERD) is a common pediatric condition that can significantly impair quality of life and lead to severe complications in medically fragile patients. Although conservative and pharmacological therapies are effective in most cases, some children require surgical intervention, which remains associated with non-negligible postoperative morbidity and the need for reoperation. This study aimed to evaluate the surgical management of pediatric gastrointestinal reflux in a tertiary referral center, focusing on surgical approaches, operative outcomes, postoperative complications, and the need for reintervention. In addition, factors associated with unfavorable surgical outcomes were investigated in order to identify potential areas for improvement in the management of medically fragile pediatric patients.

**Methods:**

A retrospective observational study was conducted on pediatric patients undergoing surgical treatment for GERD, dysphagia, or both conditions at a tertiary pediatric center between December 2016 and December 2025. Demographic characteristics, comorbidities, nutritional status, ASA score, surgical procedures, operative times, postoperative complications, and reinterventions were analyzed. Associations between baseline risk factors and postoperative outcomes were explored using Fisher's exact test. Statistical significance was set at *p* < 0.05.

**Results:**

A total of 67 patients were included in this study. The study population was characterized by a high prevalence of neurological impairment (79.1%), severe malnutrition (59.7% with weight percentile <3rd percentile), and elevated anesthesiologic risk (78.8% classified as ASA 3–4). Postoperative complications occurred more commonly among patients with severe malnutrition and high ASA scores.

**Conclusions:**

Pediatric patients undergoing surgical treatment for GERD represent a highly complex and medically fragile population. Although fundoplication remains an effective therapeutic option, postoperative complications and reinterventions continue to occur, particularly among patients with severe malnutrition and elevated anesthesiologic risk. The results of this study support the need for less invasive therapeutic strategies and further research into innovative approaches aimed at reducing the surgical burden in this high-risk population. Emerging evidence regarding autologous micro-fragmented adipose tissue therapies may offer a promising regenerative approach to reinforce lower esophageal sphincter function while minimizing surgical and anesthetic burden.

## 1 Introduction

Gastroesophageal reflux disease (GERD) is a condition in which gastric contents pass into the esophagus, leading to troublesome symptoms or clinical complications (1, 2). Physiological gastroesophageal reflux generally resolves spontaneously by 12 months of age, with only about 5% of infants remaining symptomatic (1, 3).

The clinical presentation of GERD varies significantly with age. In neonates and younger children, symptoms are often nonspecific and include excessive crying, irritability, sleep disturbances, and feeding refusal (2, 3). In severe cases, it may lead to failure to thrive due to significant caloric loss or food aversion (1, 3). In older children and adolescents, the presentation more closely resembles that of adults, with heartburn, epigastric pain, and regurgitation reported (4). Extraesophageal manifestations may also present, from chronic respiratory symptoms such as persistent cough and wheezing, to oral findings such as dental erosion and halitosis, to more severe presentations such as bilious vomiting and hematemesis (1–3). Complications are heterogeneous, including esophagitis, recurrent aspiration pneumonia, and Barrett's esophagus (1, 2).

Several risk factors can contribute and predispose to GERD development in children. Prematurity and neurological impairments, such as cerebral palsy, are closely associated with GERD in children, where the incidence of severe GERD is significantly higher due to hiatal hernia and chronic constipation. GERD is also associated with disorders affecting esophageal motility and structural anomalies such as corrected esophageal atresia and intestinal malrotation (1, 2).

GERD management in children follows a gradual approach, transitioning from conservative measures to pharmacological and surgical interventions (2, 5, 6). Non-pharmacological strategies, such as adjusting feed volumes, represent first-line treatment, especially in infants (6, 7). Pharmacological intervention is indicated for documented reflux esophagitis or where conservative measures fail (1, 6). Several treatments options exist, primarily acid suppressive therapies (proton pump inhibitors), histamine-2 receptor antagonists, and potassium-competitive acid blockers, a newer class of agents currently under evaluation in clinical trials (5–10).

Given the limited benefit of acid suppression in GERD in infants, prokinetics have been explored as an alternative; however, their efficacy as monotherapy remains limited in patients without motility disorders (6, 7). Alginates represent a safe adjunct by forming a physical barrier to reflux, effectively reducing postprandial symptoms (3, 6, 10). In refractory cases involving gastric dysmotility or excessive transient lower esophageal sphincter relaxations, agents such as the gamma-aminobutyric acid (GABA) B-type receptor agonist baclofen may be considered (5–7, 11, 12). Surgical intervention is reserved for refractory or complicated GERD, such as cases with life-threatening complications, recurrent aspiration pneumonia, or failure to thrive. Laparoscopic Nissen fundoplication remains the standard surgical procedure (2, 5, 6). In extreme cases, such as those with severe neurological impairment or multiple failed fundoplications, total esophagogastric dissociation represents a method of preventing fatal aspiration (1, 13).

This study retrospectively investigated experiences of managing patients with GERD and those with gastroesophageal reflux following percutaneous endoscopic gastrostomy (PEG) tube placement at a tertiary children's hospital, including surgical techniques, operative time, postoperative outcomes, and hospital length of stay (LOS). The prospects of innovative non-surgical approaches representing less invasive options, particularly for children with anesthesia contraindications for reflux surgery or relapse after reflux surgery, were also explored.

## 2 Materials and methods

### 2.1 Study design

Clinical data were collected retrospectively from the medical records of patients who presented with GERD, dysphagia, or both conditions between December 2016 and December 2025 at our institute. The extracted variables included demographic characteristics (age, sex, height, weight, body mass index [BMI], weight percentiles), comorbidities at admission, and surgical details (technique, whether fundoplication or gastrostomy was performed, operative time, age at surgery, American Society of Anesthesiologists [ASA] physical status [anesthesia risk], hospital LOS, postoperative outcomes).

Although data on hospital LOS were collected, these were excluded from analysis as they were often influenced by patients’ transfer to other hospital units for ongoing management of associated comorbidities. Although height, BMI, and their corresponding percentile data were collected, the analysis focused exclusively on weight-related parameters.

### 2.2 Inclusion and exclusion criteria

To ensure a homogeneous study cohort, reliable data collection, and a comprehensive and accurate analysis of the outcomes, eligibility criteria were considered during patient enrollment. The inclusion criteria were as follows: aged <18 years at the time of enrollment; a clinical or instrumental primary diagnosis of GERD, dysphagia, or both; and no history of prior gastrointestinal (GI) surgical intervention. Exclusion criteria included any surgical indication unrelated to GERD or dysphagia; a history of previous GI surgical intervention; an age >18 years at the time of enrollment; and incomplete medical records/insufficient clinical data.

### 2.3 Diagnosis and follow-Up

At our institution, gastroesophageal reflux is diagnosed by an x-ray contrast study of the upper GI tract. In case of discrepancy between symptoms and imaging, an esophagogastroduodenoscopy with intestinal biopsy and 24 h pH-impedance testing are performed. Postoperatively, patients who exclusively undergo PEG tube placement are recommended a repeat x-ray contrast study of the upper GI tract only if they develop symptoms suggestive of gastroesophageal reflux. Patients who undergo fundoplication of the stomach with or without PEG placement usually repeat the x-ray contrast study after 6 months.

### 2.4 Primary and secondary endpoints

The primary endpoint was the clinical response to surgical treatment in patients with GERD or development of gastroesophageal reflux following the initial operation. For this study, clinical response was defined as a multidimensional outcome encompassing treatment efficacy and perioperative safety. The secondary endpoint was to identify potential areas for improvement in GERD or postoperative gastroesophageal reflux management, particularly in medically fragile patients, aiming to reduce the need for major surgical procedures.

### 2.5 Statistical analysis

All statistical analyses were performed using the open-source software Jamovi (version 2.7.29); *p*-values < 0.05 were considered statistically significant. The baseline characteristics of the study cohort were summarized using descriptive statistics. Categorical variables are expressed as absolute frequencies (n) with percentages (%). Continuous variables are expressed as medians with interquartile ranges (IQRs) if non-normally distributed or means ± standard deviations if normally distributed. To prevent distortion of standard surgical metrics, outliers identified by visual inspection using boxplots were excluded from the mean calculations using selective mathematical filtering. Associations between categorical variables were assessed using Fisher's exact test, given the small sample size and low expected event counts within contingency table cells which would have compromised the validity of a standard Pearson's chi-square test.

The primary endpoint was addressed by evaluating the impact of the primary surgical approach (isolated gastrostomy vs. combined gastrostomy and fundoplication) on the development of postoperative gastroesophageal reflux in patients with isolated dysphagia. The secondary endpoint was addressed by investigating the association between baseline clinical characteristics, specifically preoperative nutritional status (weight percentile below 3rd), anesthesiologic risk stratification (ASA physical status 1–2 vs. 3–4), and the incidence of postoperative complications. Inferential analyses comparing postoperative complication rates between patients undergoing primary fundoplication and those requiring salvage reinterventions were not performed, as the limited sample size in the reintervention subgroup precluded achieving adequate statistical power. Therefore, these outcomes were analyzed as descriptive clinical trends.

## 3 Results

### 3.1 Population demographics and clinical characteristics

The study population comprised 67 pediatric surgical patients (27 females, 40.3%; 40 males, 59.7%). Patients were monitored throughout the initial surgical procedure and postoperative follow-up, with particular attention to postoperative outcomes and the need for reintervention.

The age distribution varied by the number of surgical interventions. Patients undergoing their primary operation ranged from 0 to 200 months (median: 59 months; IQR: 14–161). The 14 patients who developed postoperative complications and required surgical reintervention ranged from 3 to 216 months (median: 50 months; IQR: 22–129). Two patients (aged 4 and 31 months) required a third procedure. One patient experienced further refractory complications and underwent a fourth surgical intervention at 60 months of age.

To better evaluate the prevalence of comorbidities across the cohort, patients were divided into four categories (Table 1). Of the total 67 patients, 65 (97.0%) had at least one comorbidity, with only 2 (3.0%) having none. Neurological impairment was the most common observed comorbidity (*n* = 53, 79.1%), followed by genetic disorders (*n* = 30, 44.8%), anatomical malformations of the GI tract (*n* = 14, 20.9%), and other conditions (*n* = 1, 1.5%).

**Table 1:** Comorbidities prevalence in our study population.

Comorbidities	*n*	%
Neurological Damage	53	79.1%
Genetic Disorder	30	44.8%
GI Anatomical Malformation	14	20.9%
Others	1	1.5%
At least one comorbidity	65	**97**.**0%**

GI, Gastrointestinal.

### 3.2 Surgical procedures

To allow for detailed analysis of the surgical interventions, patients were stratified by the indication for their initial procedure into three diagnostic categories: GERD (*n* = 13, 19.4%), GERD associated with dysphagia (*n* = 23, 34.3%), and isolated dysphagia (*n* = 31, 46.3%). The prevalence of fundoplication, gastrostomy, and combined gastrostomy with fundoplication was evaluated for each subgroup. Of the 13 patients with GERD, 12 (92.3%) underwent fundoplication and 1 (7.7%) underwent a combined PEG tube placement and Nissen fundoplication procedure. Of the 12 patients treated with fundoplication alone, 9 (75.0%) underwent Nissen fundoplication and 3 (25.0%) underwent Thal fundoplication. Of the 31 patients with isolated dysphagia, the initial procedure was PEG placement alone for 28 (90.3%), combined PEG placement with Nissen fundoplication for 1 (3.2%), isolated Dor fundoplication for 1 (3.2%), and Heller esophageal myotomy combined with Dor fundoplication for 1 (3.2%). Of the 23 patients with both GERD and dysphagia, only 1 (4.3%) underwent gastrostomy with PEG tube placement alone; the remaining 22 (95.7%) underwent combined PEG tube placement and Nissen fundoplication.

### 3.3 Operative time

The mean operative time for isolated PEG tube placement was 25 ± 14 min. Regarding anti-reflux procedures, mean operative time was 139 ± 38 min for Nissen fundoplication and 145 ± 19 min for Thal fundoplication. Dor fundoplication, performed in a single patient, required 205 min. The mean operative time was 147 ± 33 min for combined PEG tube placement and Nissen fundoplication; Heller esophageal myotomy combined with Dor fundoplication, performed in a single patient, required 120 min.

The mean operative times for secondary procedures were 27 ± 5 min for isolated PEG tube placement, 90 ± 28 min for Nissen fundoplication, and 57 ± 20 min for PEG with jejunal extension tube placement.

Only two patients (3.0%) required a third intervention. The operative time was 133 min for an isolated Nissen fundoplication and 135 min for a combined PEG tube placement and Nissen fundoplication. Only one patient (1.5%) underwent a fourth surgical procedure, a Nissen fundoplication, which took 137 min.

### 3.4 Postoperative complications

Overall, 14 patients (20.9%) experienced complications following the initial procedure and consequently required reoperation. Complications were observed in four patients with GERD, four with combined GERD and dysphagia, and six with isolated dysphagia. Following the first surgery, gastroesophageal reflux occurred in five patients (7.5%): four underwent isolated PEG tube placement and one underwent Thal fundoplication. All five patients subsequently underwent Nissen fundoplication. Other complications included buried bumper syndrome (*n* = 2, 3.0%), gastric feeding intolerance (*n* = 2, 3.0%), PEG tube dislocation (*n* = 1, 1.5%), difficulty swallowing (*n* = 1, 1.5%), food aversion (*n* = 1, 1.5%), gastroparesis (*n* = 1, 1.5%), and impaired gastric emptying (*n* = 1, 1.5%). These complications and their distributions are outlined in Table 2*.* Following the second surgical intervention, 12 of 14 patients (85.7%) experienced no further postoperative complications. One patient (7.1%) developed recurrent gastroesophageal reflux and another (7.1%) developed gastroesophageal reflux associated with recurrent hiatal hernia.

**Table 2:** Distribution of postoperative complications.

Complications	*n*	%
Gastroesophageal reflux	5	**7**.**5%**
PEG dislocation	1	1.5%
Buried bumper syndrome	2	3.0%
Difficult swallowing	1	1.5%
Food aversion	1	1.5%
Gastric feeding intolerance	2	3.0%
Gastroparesis	1	1.5%
Impaired gastric emptying	1	1.5%
None	53	**79**.**1%**

N, number; PEG, Percutaneous Endoscopic Gastrostomy.

The need for subsequent reinterventions was limited: only two patients required a third surgical procedure, with one undergoing isolated Nissen fundoplication and the other undergoing a combined procedure. Of these patients, one remained free of complications and the other developed recurrent gastroesophageal reflux. One patient underwent a fourth Nissen fundoplication, after which no postoperative complications were observed.

### 3.5 Postoperative gastroesophageal reflux in patients with isolated dysphagia

Of the 31 patients with isolated dysphagia, gastroesophageal reflux developed postoperatively in 3 (10.7%) of those treated with PEG tube placement alone. Notably, the primary surgical approach (isolated gastrostomy vs. combined gastrostomy and fundoplication) was not significantly associated with the onset of postoperative gastroesophageal reflux in patients with isolated dysphagia (*p* = 1.000, Fisher's exact test). All three patients who developed gastroesophageal reflux following PEG tube placement subsequently underwent secondary Nissen fundoplication.

### 3.6 Surgical risk stratification

#### 3.6.1 Weight percentiles

In total, 40 patients (59.7%) had a weight below the 3rd percentile and 27 (40.3%) had a weight at or above the 3rd percentile. For these 27 patients, the distribution of weight percentiles ranged from the 3rd to the 96th percentile (median: the 32nd percentile). The interquartile analysis demonstrated that 75.0% of patients had a weight below the 46th percentile.

Potential associations between baseline nutritional status and the overall incidence of postoperative complications were also investigated (Table 3). Postoperative complications occurred in 11 patients with a weight below the 3rd percentile and 3 patients with a weight at or above the 3rd percentile. Thus, the complication rate was considerably higher in patients below the 3rd percentile (27.5%) than those at or above the 3rd percentile (11.1%); however, this difference did not attain statistical significance (*p* = 0.134, Fisher's exact test).

**Table 3:** Complications according to nutritional status.

Weight percentile	Complications	
	*N* (%)	Y (%)
Weight Percentile > 3rd	24 (88.9%)	3 (11.1%)
Weight Percentile < 3rd	29 (72.5%)	11 (**27.5%**)

N, No; Y, Yes.

#### 3.6.2 ASA physical status

ASA physical status was unavailable for one patient (1.5%). Of the remaining 66 patients, most had an ASA physical status of 3. This classification was the same for 12 patients requiring a second intervention, both patients who underwent a third procedure, and the single patient who required a fourth intervention.

To assess the impact of baseline ASA physical status on surgical outcomes, patients were stratified into low-to-moderate risk (status 1 or 2) and high risk (status of 3 or 4) groups. Notably, all complications occurred in the high risk group, in which the complication rate was 25.0%; this value was 0.0% in the low risk group (Table 4). However, this difference was not significant (*p* = 0.104, Fisher's exact test).

**Table 4:** Complications according to ASA score.

ASA score	Complications	
	*N* (%)	Y (%)
ASA 1–2	10 (100%)	–
ASA 3–4	42 (75%)	14 (**25%**)

ASA, American Society of Anesthesiologists.

## 4 Discussion

This pediatric cohort provides an overview of the current challenges in managing GERD and postoperative gastroesophageal reflux in a specialized setting. Our findings confirm the high success rates of standard anti-reflux procedures, consistent with prior studies, and offer new insights into the therapeutic durability of these interventions in medically fragile populations.

One finding of notable clinical interest concerns the management of pediatric patients with dysphagia and the long-term surgical outcomes. A substantial proportion of patients who underwent isolated PEG tube placement subsequently developed gastroesophageal reflux, necessitating a secondary delayed fundoplication. This mirrors the trend of pediatric patients with dysphagia initially managed solely with PEG tube placement exhibiting a susceptibility to postoperative gastroesophageal reflux requiring reoperation. Although the inferential analysis did not attain statistical significance in demonstrating the superiority of a combined anti-reflux approach to isolated PEG tube placement, this was limited by the small sample size and the low absolute number of postoperative gastroesophageal reflux events recorded during follow-up. The current evidence remains divided regarding the relationship between PEG tube placement and gastroesophageal reflux development, highlighting the need for careful evaluation of the risks and benefits associated with fundoplication when not primarily indicated (14).

Prior studies have highlighted pediatric patients with GERD as an exceptionally high-risk surgical population (2, 6, 14, 15). This systemic vulnerability is reflected in our descriptive data. Specifically, neurological impairment was the predominant comorbidity, experienced by 53 patients (79.1%); severe malnutrition was also prevalent, with 40 patients (59.7%) presenting with a weight below the 3rd percentile. Stratification by ASA physical status (anesthesia risk) confirmed this critical status: a total of 56 patients (84.9%) had an ASA physical status of 3 or 4 at the time of the initial procedure, rising to 100.0% in patients requiring subsequent reinterventions. These factors directly impact postoperative morbidity, consistent with an international study demonstrating that nutritional deficits in fragile pediatric patients increase perioperative morbidity (15). Similarly, the higher incidence of complications in patients with an ASA physical status of 3 or 4 underscores that underlying systemic frailty, not surgical technique alone, is the primary determinant of perioperative risk.

To date, the therapeutic paradigm for pediatric patients who do not respond favorably to conservative care, encompassing those with primary GERD nonresponsive to maximized medical therapy and those with dysphagia who fail intensive swallowing rehabilitation, remains significantly constrained. Where pharmacological options are exhausted, the alternative is often to escalate to invasive anti-reflux surgeries such as laparoscopic or robotic-assisted fundoplication (1, 6). While effective, these procedures are associated with a substantial burden of perioperative morbidity, anesthesia risks, and long-term mechanical complications, particularly in medically complex cohorts with neurological impairments (1, 13). Consequently, there is a compelling need to explore new, minimally invasive therapies which reinforce the anti-reflux barrier without the anatomical disruption inherent to major foregut reconstructions (16, 17).

Emerging international studies have focused on the regenerative and structural potential of autologous micro-fragmented adipose tissue (18). In pediatrics, autologous fat grafting has shown promising results for the conservative management of severe fecal incontinence secondary to anorectal malformations and cloacal anomalies, as well as in refractory complex perianal Crohn's fistulas (19–21). Building on this, recent investigations using animal models of GERD have evaluated the therapeutic potential of adipose-derived mesenchymal stem cell injection into the lower esophageal sphincter, reporting significant restoration of resting sphincter pressures, reduced gastroesophageal reflux metrics, and structural regeneration of the muscular layer at the gastroesophageal junction (22).

Conversely, historical attempts at endoscopic injection therapies using synthetic copolymers have yielded disappointing results in treating GERD. While these copolymers have been used safely for many years in managing vesicoureteral reflux and urinary incontinence, studies on their application at the gastroesophageal junction have revealed significant limitations. These include material reabsorption, lack of tissue integration, and, in some cases, severe ischemic complications due to accidental intravascular administration, leading to embolic migration within visceral arterioles (16, 17, 23). Indeed, unlike these synthetic bulking agents, autologous micro-fragmented fat could represent an ideal biocompatible substrate combining immediate mechanical augmentation with long-term results (22).

Based on this promising evidence, we recently began investigating the use of autologous micro-fragmented adipose tissue injections in managing urinary incontinence in patients with/operated on for complex congenital malformations such as bladder exstrophy–epispadias complex and cloacal malformation, with hopeful results. In addition, an innovative clinical project is being launched at our pediatric center to evaluate the safety and efficacy of intersphincteric autologous micro-fragmented adipose tissue injections targeted to control lower esophageal sphincter incontinence in pediatric patients. By implementing this minimally invasive technique, we aim to reduce the impacts of surgery and anesthesia in this fragile pediatric population. If proven effective, this regenerative approach could revolutionize GERD management in pediatric patients, promoting rapid postoperative recovery, minimizing severe complications, and establishing a viable alternative to traditional reflux surgery.

Limitations of this study include its retrospective design and relatively small cohort. Prospective studies with larger cohorts are required to validate these findings and support continued progress in the surgical management of GERD. Despite these limitations, this study reinforces that pediatric patients requiring surgical intervention for GERD or postoperative reflux often represent a highly complex and fragile population, characterized by the prevalence of severe neurological impairments, preoperative malnutrition, and high ASA physical statuses. The findings demonstrate that while standard surgical reconstructions such as traditional fundoplication remain effective therapeutic options, they carry a burden of postoperative morbidity and mechanical failure, often leading to consequential reoperations.

These clinical trends indicate that a patient's underlying systemic frailty, rather than surgical technique alone, could serve as the primary determinant of perioperative outcomes. Thus, endoluminal techniques may represent a revolutionary therapeutic perspective to optimize recovery and reduce severe long-term complications in medically fragile pediatric patients.

Overall, these findings contribute to a deeper understanding of patient-specific risk factors and support the refinement of evaluation and management methodologies. Identifying these clinical patterns is a critical step toward optimizing existing strategies and developing new treatment options, with the long-term goal of reducing the need for invasive surgical procedures in this highly vulnerable pediatric population.

## Data Availability

The raw data supporting the conclusions of this article will be made available by the authors, without undue reservation.
